# State-of-Art of Standard and Innovative Materials Used in Cranioplasty

**DOI:** 10.3390/polym13091452

**Published:** 2021-04-30

**Authors:** Valentina Siracusa, Giuseppe Maimone, Vincenzo Antonelli

**Affiliations:** 1 Department of Chemical Science, University of Catania, Viale A. Doria 6, 95125 Catania, Italy; 2 Department of Neurosurgery, Hospital M. Bufalini—AUSL della Romagna, Viale Ghirotti 286, 47521 Cesena, Italy; giuseppe.maimone@auslromagna.it (G.M.); vincenzo.antonelli@auslromagna.it (V.A.)

**Keywords:** cranioplasty, neurosurgery, synthetic cranioplasty, cranial defect, skull reconstruction, biomaterials, polymers, polymethylmethacrylate (PMMA), polyetheretherketone (PEEK), polyethereketoneketone (PEKK), polylactic acid (PLA), polycaprolactone (PCL), polyglycolide (PGA)

## Abstract

Cranioplasty is the surgical technology employed to repair a traumatic head injury, cerebrovascular disease, oncology resection and congenital anomalies. Actually, different bone substitutes are used, either derived from biological products such as hydroxyapatite and demineralized bone matrix or synthetic ones such as sulfate or phosphate ceramics and polymer-based substitutes. Considering that the choice of the best material for cranioplasty is controversial, linked to the best operation procedure, the intent of this review was to report the outcome of research conducted on materials used for such applications, comparing the most used materials. The most interesting challenge is to preserve the mechanical properties while improving the bioactivity, porosity, biocompatibility, antibacterial properties, lowering thickness and costs. Among polymer materials, polymethylmethacrylate and polyetheretherketone are the most motivating, due to their biocompatibility, rigidity and toughness. Other biomaterials, with ecofriendly attributes, such as polycaprolactone and polylactic acid have been investigated, due to their microstructure that mimic the trabecular bone, encouraging vascularization and cell–cell communications. Taking into consideration that each material must be selected for specific clinical use, the main limitation remains the defects and the lack of vascularization, consequently porous synthetic substitutes could be an interesting way to support a faster and wider vascularization, with the aim to improve patient prognosis.

## 1. Introduction

**Figure 1:**
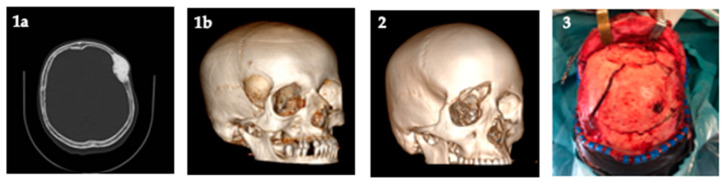
Example of causes that require cranioplasty (authors personal database images): (**1a**) axial computerized tomography (CT) scan of tumor involving cranial bone; (**1b**) 3D-CT scan of tumor involving cranial bone; (**2**) 3D-CT scan of depressed skull fracture; (**3**) surgical exposition of complex depressed skull fracture.

Cranioplasty is an old surgical procedure used to repair cranial defects, offering at the same time protective and cosmetic benefits for patients. The main causes that require cranioplasty are birth defects (absence of an intact cranial vault), infection of the cranial contents, tumor removal, decompressive craniectomies and traumatic injuries, for all age people [1,2,3,4,5,6]. This procedure can improve electroencephalographic abnormalities, cerebral blood flow abnormalities and other neurological abnormalities [1,2,3,4,5,6]. The contraindication for such procedure could be infections, hydrocephalus and brain swelling but delaying cranioplasty could cause preclusion in autograft devitalization or allograft infections. Further, in order to allow spontaneous ossification, studies reported that foreign materials should be implanted after 1 year [4]. As reported by several authors [1,2,3,4,5,6], materials used for cranioplasty have to be radiolucent, resistant to infections, non-conductive of heat or cold, malleable, mechanical resistant, ready and easy to use and of course allowed at low cost. Different materials were used over time, derived from biological sources or synthetic polymers, as reported in Figure 1:

The first material used for cranioplasty was a thin gold plate, used for covering a Peruvian skull left frontal defect, dated 2000 BC [7]. Of course, in ancient times, precious metals were used for the richest patients while gourd materials were used for less advantaged people. Then, those materials were replaced by animal bones from dogs, apes, gooses, rabbits and eagles but also horn from the ox and buffalo, and ivory. Subsequently, cartilage from cadaver was considered as cranioplasty materials, due to its malleability and resistance to infections, but due to its low resistance and low calcification its use was abandoned [7]. The cadavers were used to resect bones but also in this case the high rate of infection and bone resorption made the cadaver skull allografts a bad choice as cranioplasty material. Finally, autologous bone graft was considered and preferred in order to reduce the rejection by the host. Firstly, bones from the tibia, ilium, ribs, sternum, scapula, fascia and fat were used [7]. Subsequently, cranium bone graft becomes widespread (Müller–König procedure), by replacement of the original bone, removed during craniectomy [7]. This procedure is still particularly preferable for pediatric patients, due to the easier body reintegration [7]. Further, autologous bone could be stored by cryopreservation technology or by placement in a subcutaneous pocket placed in abdominal wall [7]. Cryopreservation can destroy the matrix where the osteoprogenitor cells enter and take root, consequently the last procedure is preferred, due also to the lower infection rate and low cost. Nonetheless, despite the benefits, a common complication could be the bone flap resorption, with a structural break-down result, with the consequent necessity of reoperation and bone replacement with other materials such as metal, ceramic or plastic [4,8]. Matsuno et al. [9] reported that a 25.9% rate of infection was recorded by the use of autologous bone graft in respect to synthetic materials such as polymethylmethacrylate (PMMA), alumina ceramics or titanium mesh. So, despite autologous bone graft is preferred for cosmetic result, its low cost and patient incorporation for storing, synthetic materials were largely considered as a good alternative to avoid complications due to bone resorption, infection, reduced strength and malleability. Over time, several materials have been taken into account. An interesting review was presented by Morselli et al. [10], where they allied heterologous materials choose to cranioplasty most frequent complications. In particular titanium, PMMA, polyetheretherketone (PEEK) and hydroxyapatite (HA) materials were analyzed, selecting retrospective and prospective studies that thought postoperative complications in custom-made reconstruction [10].

As highlighted by Morselli et al. [10], despite the great interest in cranial reconstruction procedure, there is a low number of multicentric study. Consequently, there is a broad range in the type of cranioplasty material utilized in various countries. As reported by Morselli and collaborators [10], “titanium is mostly used in Australia, the UK and Germany, PMMA in the USA, PEEK in the USA, Singapore and Korea, HA in France and Italy”.

In this contest, the study was aimed at overthrowing the state-of-the-art of all materials used for cranioplasty. In particular, the use of synthetic polymer materials in the field of medicine was emphasized by authors, motivated by the importance of making polymers and biopolymers, even more supply chain actors for osseous replacement and repair in cranioplasty. Polymers are employed in all fields and as special materials in biomedicine, due to their low market cost and appropriate chemical–physical, mechanical and barrier characteristics. Among the polymers, biomaterials have shown strong interest following the recent trends toward greener material and waste reduction. Among the plastic annual production, biopolymers represent a lower percentage but their demand is expected to growth [11,12,13,14,15]. Biopolymers can offer attractive alternatives as long as their synthesis and characterization can be easily tailored for special technological applications, as cranioplasty. The issue of sustainability is of great importance, encouraging academia, industry and politics to develop sustainable alternatives for preserving resources for future generations, focusing the attention versus biomaterials. The use of biopolymers in specific field of medicine, like cranioplasty, could be an interesting challenge. It is out of doubt that fabricating new materials requires an interdisciplinary approach, combining cellular and molecular biology with medicine, biochemistry, immunology, engineering and material sciences. The selection of the material depends on several factors related to the patient such as age, size, type and location of the cranial defect but also to other factors such as surgeon preference and costs, which is an important parameter related to the economic feasibility of the medical structure.

To our knowledge, few papers were published dealing on the “best choice” material [1,2,3,4,5,6,10,15].

Our research was conducted by introducing as keywords “cranioplasty”, “neurosurgery”, “biomaterials” and “polymers”, without any limitation on the time frame. Seventy-one documents were analyzed, particularly oriented on synthetic polymers and biopolymers, as reported in the reference session.

## 2. Short Cranium Anatomy and Cranioplasty Fixation Techniques

Brain, meninges and cerebral vasculature are sheltered by the cranium. There are eight cranial bones: two parietal, two temporal, one frontal, one occipital, one sphenoid and one ethmoid [16]. The parietal bones are located on the top and sides of the cranium while the temporal bones are on the head, under the parietal bones and above and behind the ears. The frontal bone forms the forehead, the occipital bone forms the back-base of the cranium, the sphenoid bone forms the eye orbit and lastly the ethmoid bone forms part of nasal and eye cavities [17,18].

Cranioplasty fixation techniques could be briefly summarized in wiring, suturing, plating, clamping and strips procedures [17,18]. Wiring is the simplest and most rapid technique and consists of drilling holes in each bone flap and in the adjacent skull edge, insert wires through the holes, twist together the extremities and cut-off the wires excess. Suturing techniques with inert wires, applied for many years, could result in displacement of the bone plates, resulting in depression of the flap [17,18]. Plating consists of fixing on the skull an implant (plates) made of different materials, along the perimeter of the craniotomy defect and secured it on craniotomy flap situated in the skull, by use of screws. Titanium screws of different length and titanium in the form of mini plates could be used [16]. This technique is expensive and could be a long duration in time procedure [16]. Clamping using titanium could be a suitable alternative for cranioplasty fixation, without dura mater separation from the bone, offering better cosmetic and resistant results in respect to suture and wire fixation, with a better dealing with deformities of the cranial flap. Strip technique performed by using titanium strips offers better resistance to injury from impact, if compared to other methods [17,19].

Each technique of course is related to the specific case under study and must be carefully selected, taking into consideration not only the cosmetic outcome but especially risk of infection and anesthetic complications for the patient. As an example, instead of a usual two-step procedure (resection and custom-made cranioplasty reconstruction), a single-step surgical procedure could be considered, as reported in the literature [20]. Due to high cost and time required [21], this procedure was at the beginning used for benign lesions but due to the new technology an increasing use of such approach has been developed [22,23].

## 3. Discussion on Cranioplasty Materials

### 3.1. Synthetic Materials

Materials employed to replace bone tissue should meet specific characteristics such as biocompatibility, bioresorbability, osteoconductivity, osteoinductivity, structural bone similarity, porosity and mechanical resistance. At the same time, such materials have to be easy to use, easy to shape, safe and at low cost [24]. The only material that appears to meet all those specifications is autologous bone. Its use avoids immunogenicity or rejection problems and any disease transmission risk [24]. Consequently, autologous bone graft continues to be the gold standard technique used. As reported by de Grado and collaborators [24] the most important disadvantage is certainly the comorbidity associated with the presence of a second surgical site: the donor site. Others several complications could be chronic pain (2.5–8% of cases), dysesthesia (6% of cases) and infections (2% of cases). However, currently, many different bone substitutes can be used, either derived from biological products such as demineralized bone matrix, platelet-rich plasma, hydroxyapatite, adjunction of growth factors like bone morphogenetic protein or synthetic such as calcium sulfate, tricalcium phosphate ceramics, bioactive glasses and polymer-based substitutes (petroleum-based polymers and biodegradable polymers). Being dependent on the scope, each material has his peculiar characteristics to be considered [24].

Bone replacement can be due to several causes such as infection, tumor, trauma, surgery, congenital etiology and so on.

The main interest is versus materials with a structure suitable for a faster and wider vascularization. So, instead of autogenous or allogenous, allogenic and xenogenic bones, substitutes bones such as synthetic, inorganic or biologically organic combination can be considered. Allogenous, allogenic and xenogenic bones pose some limitations such as virus transmission, high cost, immunogenicity problems and disease transmission (porcine endogenous retrovirus (PERV) and the bovine spongiform encephalopathy (BSE)). In order to avoid such limitations, the use of synthetic bone, biological or not, is becoming even more popular [24].

**Figure 2:**
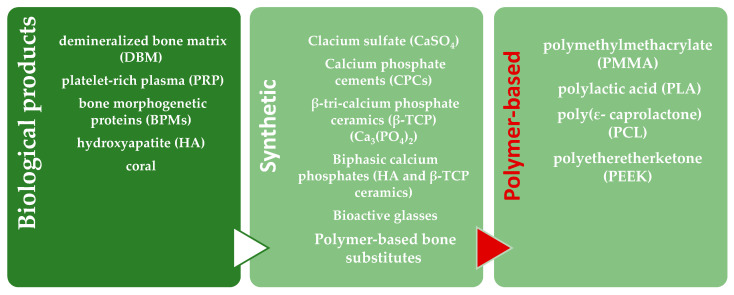
Bone substitutes categories.

As reported in Figure 2, bone substitutes can be classified into two main categories: those derived from biological sources and synthetic ones:

The synthetic category includes the polymer-based bone substitutes, particularly highlighted in this review.

Over the years, various materials have been considered and examined to be adapted to our body.

### 3.2. Bones Substitutes Derived from Biological Sources

Demineralized bone matrix (DBM): By acid-treatment, the mineral matrix is removed but the organic matrix and growth factors (morphogenetic protein (BMP), insulin growth factor (IGF), transforming growth factor (TGF) or fibroblast growth factor (FGF)) are preserved. In general, it is formed prevalently by collagen, with a 5% of growth factors, which favors osteoinductive capabilities and osteoconductive properties [24]. Nevertheless, during processing, the osteogenic capacity is lost. Due to the demineralization process by acid, no immunological rejection was recorded, due to the absence of the antigenic surface structure, which was damaged during the treatment. DBM is derived from human bone and in respect to iliac crest bone autograft is more expensive, lower mechanical performance that allows one to use it only for filling purposes and not as stand-alone bone substitute. DBM is actually used in 50% of allografts performed in the United States [24].

Platelet-rich plasma (PRP): It is obtained from the patient’s blood and used as a gel. The blood platelets, extracted by centrifugation, are combined with thrombin and calcium chloride [25]. Despite PRP reach in platelet derived growth factors (PDGF), vascular endothelial growth factor (VEGF), IGF and TGF could be used for promoting bone regeneration and showing limited infectious risks and adverse effects, it has low mechanical resistance and could not be used as a stand-alone bone substitute while it is used as a supplement for other materials [24,26].

Bone morphogenetic proteins (BPMs): BPMs are growing factors produced by osteoblasts. They are involved in the skeletogenic process, in the ectopic bone formation, playing a key role in the recruitment of osteoprogenitor cells in bone formation sites. Recombinant human BMPs, named rhBMP-2 and rhBMP-7, were synthetically obtained, approved by the Food and Drug Administration (FDA) for clinical use. The adverse effects could be paradoxical inhibitory effects, heterotrophic ossification, osteolysis, infection and retrograde ejaculation [24]. Despite those negative effects, it seems promising in nonunions resolutions [27].

Hydroxyapatite (HA): It is the major mineral component of teeth and bones. Its chemical formula is Ca_10_ (PO_4_)_6_(OH)_2_, as crystalline hexagonal lattice. Due to its porosity, it presents osteoconductive properties, with a high degree of tissue ingrowth and vascularization [28]. HA alone shows slow resorption and consequently it could be maintained at least to 3 years after implantation, with a painful bone ingrowth and cell colonization progress. Due to its mechanical performance such as brittleness, low tensile strength, with compression resistance up to 160 MPa, it is used for small bone defect where a lower loading is required [28]. In order to enhance its quality, HA is not used alone, consequently natural and synthetic forms are preferred, such as tricalcium phosphate HA (HA-TCP) and in composite with collagen, enhancing osteoblasts differentiation and accelerating osteogenesis. For HA–collagen composites it was observed that the ductile properties of collagen could increase the fracture toughness of HA [28].

Coral: Its use, as a bone substitute, has been approved by the FDA in 1992. In general, it is transformed industrially into HA, giving coralline-HA as granules or blocks. It can be invoked as a growth factor carrier, such as BMP, TGF, and/or FGF. It is osteoconductive, with good bone-bonding capacity and low capacity to promote disease transmission or deep infections [29].

### 3.3. Synthetic Inorganic Bones Substitutes

Calcium sulfate (CaSO_4_): It was accepted as a bone substitute from the FDA in 1996. It has a similar bone structure, inexpensive and available as hard pellets and injectable fluids forms. It is osteoconductive and resorbs in 1–3 months, creating porosity while having bony ingrowth [24].

Calcium phosphate cements (CPCs): It was approved by FDA in 1996 and consist of a calcium phosphate powder that when mixed with a liquid, forms a workable paste, with an isothermic hardening reactions that vary from 15 to 80 min, depending on the formulation [30]. It is possible to shape the paste to the bone cavity, filling all the gaps between the bone and the implant. It is brittle but it can remain in the body for up to 2 years, without resorption [24].

β-tri-calcium phosphate ceramics (β-TCP): The chemical formula is (Ca_3_(PO_4_)_2_) and it is considered the gold standard material for synthetic bone. It is biocompatible, bioresorbable due to the fact that its properties are similar to those of the bone inorganic phase. Depending on the processing, the porosity could change, changing consequently its osteoconductive characteristic. By osteoclasts, its resorption is slower (13–20 weeks) but with a total replacement by remodeled bone. In fact, as reported by Gunzburg and collaborators [31], due to its pores structure, it facilitates the colonization of osteogenic cells and nutrients, with enhanced capillarity. Despite its lower infection and nonunion complications and suitable mechanical properties, inferior to those of cancellous bones and bone allograft, it is used selectively [31].

Biphasic calcium phosphates (HA and β-TCP ceramics): Often HA and β-TCP are utilized together, due to their respective characteristics. Resorption of β-TCP is faster than those of HA but the mechanical properties of HA (average compressive resistance of 160 MPa) are slightly better than of β-TCP (average compressive resistance of 100 MPa) [24]. Their combination enhances a faster and higher bone ingrowth rate, offering at the same time a better mechanical performance. Both are osteoconductive, biocompatible, safe, non-allergenic and promote bone formation [24].

Bioactive glasses: Are silicates (SiO_2_) coupled with other minerals present in the body such as Ca, Na_2_O, H and P. In general, the composition of bioglass is: 45% silica (SiO_2_), 24.5% calcium oxide (CaO), 24.5 sodium oxide (Na_2_O) and 6% phosphorous pentoxide (P_2_O_5_) (*w*/*w*) [32]. Phosphate- and borate-based bioglass have been recently developed thanks to their easy manufacturer. Their utilization is selective, due to their brittleness and low mechanical strength and fracture resistance [32]. They have to be used in association with other bone substitutes.

### 3.4. Others Synthetic Materials

Metals: From the early 1900s, metals were largely experimented in medicine due to their easily sterilization procedure, malleable and strong features. Aluminum (Al) was the first metal but was subsequently abandoned due to infections, irritation on surrounding tissues, seizures and slow disintegration. Gold (Au) was seen as a suitable candidate due to the lower tissue reaction but was not used due to high cost and softness. Silver (Ag) became popular in 1903 but was not utilized due to the silver oxide reaction with the tissues and also too soft and unable to withstand trauma [1,2,3,4,5,6]. These metals were substituted by tantalum (Ta) due to its properties such as resistant to tissue reaction, corrosion, infection, inert and nonabsorbable. It was also abandoned due to its difficult and expensive production and its high temperature conduction, the cause of patient headaches [1,2,3,4,5,6].

Today, most metallic fixation systems are made on titanium (Ti) or cobalt-chromium [33] (Vitallium; Howmedica, Rutherford, NJ, USA), which is tissue compatible, corrosion resistant and chemically inert. However, due to their stress shielding, roentgenographic scattering, secondary devascularization, contour deformities and restriction, bioresorbable osteofixation systems have been studied, to facilitate and improve a rapid fracture healing.

Titanium: It could be used alone or in combination with other materials in order to increase the extraordinary strength and malleability. To improve its strength, it is usually manufactured as an alloy with small amounts of other metals, such as aluminum or vanadium [34]. It presents several advantages such as low risk of infection, at a rate of 2.6% in respect to the other materials used for cranioplasty [9,28], it is non corrosive and noninflammatory and it provides very good cosmetic results. Further, due to the latest 3D computer-assisted technology, it is possible to model excellent titanium mesh implants in a number of different structural forms, also for large cranial defects [35]. Those Ti mesh are placed over the defect and secured to the surrounding bone by screws. For large cranial defect, bulky pieces of curved Ti mesh are available, simplifying the precision of the adaptation process. Due to the perforated nature of the mesh, with a large number of holes, vascular ingrowth to occur from either surface is promoted. Its role today is more limited but is still selectively utilized in the older cranial defect patient where implantation times are shorter. It is considered a bioactive metal, with great potentiality for osseointegration, with appropriate porosity and texture. However, titanium plate is porous and does not involve a surface adequate for tissue ingrowth [36].

Ceramics: Those materials are used for cranioplasty due to their strength and aesthetic benefits, hardness like a diamond, chemically stable and compatible with human tissues, like acrylics materials. In order to make the ceramic radiopaque, yttrium is added. The disadvantage is that ceramics are expensive, must be preformed before the cranioplasty operation and due to their hardness they can shatter [4]. Alumina was the first ceramic used in cranioplasty due to its strength and chemical stability but alumina implants are expensive and susceptible to failure.

### 3.5. Synthetic Polymers

Natural, synthetic and biodegradable polymers planting systems are actually of great interest by surgeons due to the possibility to solve problems related to the use of glass, ceramics or metal, described above. Their different features could help the surgeon to fully understand the appropriate intraoperative application and their postoperative behavior.

Natural polymers such as collagen, alginate, agarose, hyaluronic acid derivatives, chitosan and fibrin glue have found application in non-load bearing locations [37]. Chitosan scaffolds, which is biodegradable and biocompatible, show weak mechanical resistance. To overcome these deficiencies, incorporation of other substances such as calcium phosphate or hydroxyapatite were investigated, creating composite materials.

Synthetic scaffolds, made of synthetic polymers, are actually the surgeon’s choice, especially when a structural integrity is required if used in load bearing applications. The most commons are poly(methylmethacrylate) (PMMA) and polyetheretherketone (PEEK) [1,2,3,4,5,6]. Another interesting synthetic material is polyethylene (PE). In particular, porous PE (Medpor; Porex Surgical) is commonly used [1,2,3,4,5,6].

**Figure 3:**
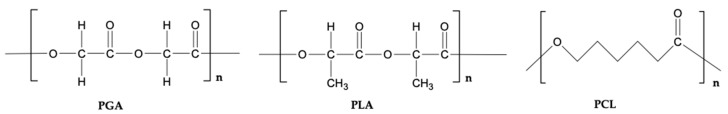
Chemical formulas of resorbable biodegradable polymers most commonly used in medicine: polyglycolic acid (PGA), polylactic acid (PLA) and polycaprolactone (PCL).

In addition to scaffold synthetic materials, surgeons are beginning to experiment with biodegradable plating systems. Their availability is growing and their efficacy, safety and cost. Ideal biopolymers for surgical application have to meet several features: they should be biocompatible with the surrounding tissue without inflammatory response, radiolucent for an easy evaluation by radiographic technique, easy to shape and mold, high volume retention rate after long term implantation, osteo-active with bone replacement at an equal rate of biomaterial resorption and readily available [37]. Most common biodegradable polymers used in medicine are those belong to the aliphatic polyesters’ families, used as implant devices. In particular they are alpha-hydroxy acids as polyglycolide (PGA), polylactides (PLA), polycaprolactone (PCL) and their copolymers [37]. Chemical formulas are reported on Figure 3:

According to the chemical structure, they should present hydrophilic (more rapid degradation) or hydrophobic (less rapid degradation) behavior, such as PGA and PLA respectively. In order to consider both characteristics, copolymer materials could be utilized. Each different polymer and copolymer present specific resorption profile. As an example, a copolymer made of 82% of PLA and 18% of PGA (LactoSorb) could be used to produce a workable fixation device for craniofacial bone, which maintain its strength for 6–8 weeks, with a complete resorption at one year after implantation [38]. Their chemical composition, morphology, crystalline versus amorphous phase, their molecular weight and their polymer processing methods play a crucial role in their mechanical performance [38]. To overcome the different mechanical resistance in respect to metal, more material (thicker or wider) could be used in a resorbable plate [33]. The implanted multimaterial devices can degrade by a two-step process: hydrolysis and metabolism. Hydrolysis begin after placement, over the ensuing months, with separation of polymer chains and consequently losing of resistance to external stresses, mass loss and resorption process. Oligomers (with lower molecular weight) and monomers are formed until much later. The second step is the digestion of monomers in body cells, with subsequent degradation and conversion in carbon dioxide (CO_2_) and water [33]. This two-phase process could be modified by choosing the correct polymer/copolymer composition and manufacturing process. Fixation methods, and consequently materials characteristics, have recorded an evolution during the last two decades [39]. In 1994, a short 1 mm thick PLA-PGA plates with standard geometry, were used for cranial vault reconstruction, with 1-mm titanium screws for fixation. The biodegradable polymer plate is completely resorbed by the body, without any adverse reactions. Since 1996, resorbable screws of 1.5 mm were also manufactured and started to be commercialized [39]. Further, a single large holed sheet or panel of resorbable polymer material was introduced, due to the possibility to cut it in any shape, during surgical operation [39]. Such a polymer panel, after warming in order to reduce accident cracking and weakening, could be cut with scissors. Single 50 mm × 50 mm panel could be sufficient for an entire cranial vault reconstruction. Small pieces of those panel could be used also to fill bone defects or to connect different panels, using sutures running through the bone piece to the holes of the panel, creating a composite bone graft [39]. For a better cosmetic result, for sagittal and occipital reconstructions, long-double row plates are utilized [39]. The effort to remove the two steps necessary for the screw placement, with wrist turning, defined as “push-in” device, is the use of the “pull-back” rivet type. Another interesting approach is to fix biomaterials plates to the cranial vault by using liquid adhesives (glue fixation method) [39].

An overview of synthetic polymers is reported.

#### 3.5.1. Poly(methylmethacrylate) (PMMA)

**Figure 4:**
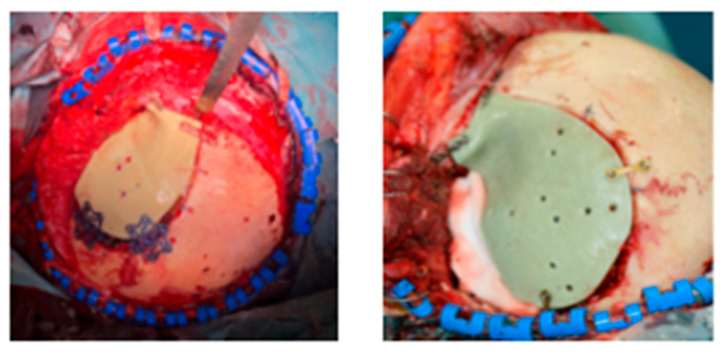
Cranioplasty in PMMA with titanium fixation plates and screws.

**Figure 5:**
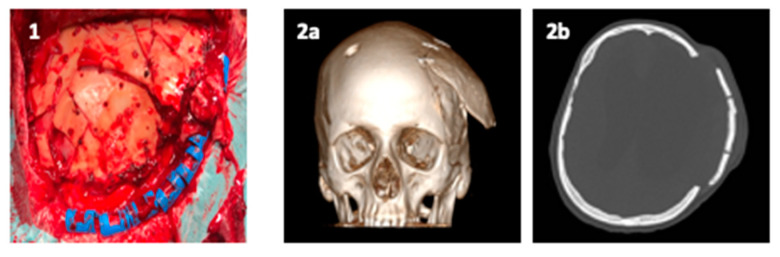
(**1**) PMMA cranioplasty fractures; (**2a**) 3D and (**2b**) axial CT-scan of PMMA cranioplasty displacement.

PMMA, a polyester obtained from acrylic acid polymerization, was discovered in 1939 and introduced in the medicine field in 1970 [40]. It was used in medicine due to the comparable bone strength, with good results to compression and torsion testing, low cost and readily available. It is considered a material better than metal because it is strong, heat resistant, radiolucent and inert. Its radiolucency characteristic is positive for the detection of the cerebral vasculature by the angiography technique but fractures of the plate became difficult to be detected [40]. To overcame this problem, barium is infused within the plate, detectable by radiographic means. PMMA shows better compression and stress resistance than HA. When associated with titanium, used as support wire mesh for the placement of large cranioplasties, a reduction in fracture was detected [41] and a more cosmetic resolution. Despite those advantages, PMMA shows high risk of extrusion, decomposition and infection (5%) [28,37]. A high rate of infection (23%) was observed in patients with a previous infection in the reconstruction region [37]. It is fabricated intraoperatively by mixing a liquid monomer with a powdered polymer. Liquid and powder could be mixed in an open bowl or using a mixing pack made of two separate chambers, one with the powder and one with the liquid. Mixing is performed by removing the central strip in which the liquid is rapidly drawn into the powder. The pack is subsequently cut and the resin is squeezed onto the application site, without off odors, hand-contoured and allowed to harden. PMMA generally adheres to bone but sometimes could be necessary to use small screws and/or plates of material skull anchorage [20,34], as reported in Figure 4:

The residual monomer, which could be coming-out from cold polymerization, is toxic. Further, the preparation of the malleable paste is an exothermic reaction (as high as 80 °C, 8–10 min), which could cause burn injuries like thermal necrosis and inflammation of the surrounding tissue [34]. Sometimes, to protect tissue from the heat, gauze saturated with saline solution are placed between the acrylic resin and the dura tissue. A very interesting scheme of PMMA paste preparation was published by Shah and collaborators [1]. PMMA is modelled by the surgeon to form a plate that then, through drilled holes, it is wired over the cranial defect. Inadequate cooling can cause damage to the brain tissue or dura in the surrounding area [18]. The surface texture ranges from smooth to coarse, with a degree of porosity ranging from 0% to 40% [42]. In respect to Ti, with an elastic modulus of approximatively 110 GPa, PMMA shows an elastic modulus of 3 GPa, with consequently less stress shielding and less loosening of fixation devices over time [42]. Examples of PMMA complication are fracture of cranioplasty and cranioplasty displacement, reported in Figure 5:

For larger defects, thin Ti mesh could be used as “floor” before adding the resin. The impact strength is improved and the polymer can well adhere and fuse during the polymerization process, creating a composite metal–plastic solid unit [34].

An interesting composite material made of bis-glycidyl methylmethacrylate, bisphenol, trethylene glycol dimethylacrylate monomer and bioactive glass [16], named Cortoss (Orthovita, Malvern, PA, USA), is actually used for skull reconstruction due to its similarity to bone and lower incident of inflammation in comparison to PMMA alone [43], despite very limited clinical data are currently available. Interesting data about the use of PMMA are reported in literature [44,45].

#### 3.5.2. Polylactic Acid (PLA)

PLA is a natural, biodegradable polyester obtained by polymerization of chiral molecule present in two stereoisomeric forms, namely D- and L-isomer, which are semicrystalline and relatively biodegradable [33,46,47]. The L-form shows a high crystallinity and consequently high strength and long degradation time. The racemic combination of both isomers, gives out an amorphous more biodegradable material, with lower strength and is clear. Being dependent on the racemic mixture ratio, the mechanical performance and the biodegradation rate could be modulated [47]. Combining the L- and D,L-lactide is feasible to obtain a copolymer with retention of mechanical strength for 6–9 months and resorption in 24–36 months [47]. Strength and degradation features depend not only on the crystalline/amorphous phase ratio but also on the degree of polymerization, associated with the intrinsic viscosity and consequently to the molecular weight of the copolymer [47]. The choice depends on the surgeon’s preference and clinical application. For example, in children it is important that the material resorbs much faster than in the adult [33]. If the patient goes into radiation therapy, the implant must stay longer to allow better bone healing. Many researchers have devoted their study to producing biomaterial used in skeletal fixation of the craniofacial skeleton, taking into consideration the two different characteristics of the produced biomaterial: longevity and mechanical strength [48]. The first copolymer of L-lactide and glycolide was approved by FDA in 1996, named LactoSorb (LactoSorb, W. Lorenz, Jacksonville, FL, USA), with the PLA homopolymer in the L-form [48]. The ratio L-lactide:glycolide monomers was of 82:18, with a degradation of mechanical strength to approximately of 70% by 6–9 weeks and complete resorption by 12 months [48]. On 1998, a copolymer of 70% of L-lactide monomer and 30% of D,L-lactide monomer, named 70:30 DLLA polymer, was approved by the FDA for medical application (MacroPore, MacroPore Biosurgery, Inc., San Diego, CA, USA) [48]. Considering that the glass transition temperature is of 55 °C, heat could be utilized to mold this implant. Generally, the template is formed in water at 70 °C. At this temperature the polyester becomes soft in a few seconds, could be molded, cooled in the desired shape and could be placed in the patient. As reported by Habal [48], DLLA material retains approximately 70% of its initial strength after 9 months and approximately 50% after 12 months, with resorption completed by 24–36 months.

Other resorbable polyesters from Bionx, Leibinger (delta system and the new delta system); Synthes (resorbable system); KLS Martin (resorb-X) and Inion (two systems) are all FDA-approved and available for cranioplasty use [48]. The differences among these systems are the ratios of the copolymers employed in the compositions, which affect their longevity, a factor of great importance to surgeons. This longevity is associated with the ester linkage scission, with H_2_O sorption from the body, with a reverse process of lactic acid polymerization, where ester bonds are formed and H_2_O released. The hydrolysis of lactide implant (homopolymer or copolymer) continues until the last lactic acid molecule is released, which is then metabolized into glucose or into CO_2_ and H_2_O (Krebs tricarboxylic acid cycle) [48]. There are several factors that can influence the sorption process by the body. Higher is the intrinsic viscosity and consequently the molecular weight, longer is the resorption time. Further, larger in size is the lactide implant more time is required before the implant resorption can be completed. Higher polymer crystallinity leaves less space for H_2_O access, retarding the resorption time, facilitated instead in an amorphous phase. Implant porosity, increasing the surface area, facilitate the H_2_O access, decreasing consequently the resorption time [48]. The mechanical performance, as the tensile strength (about 30% of the strength of bone, which is about 82 MPa, as reported in the literature [49], remains near 100% at 3 months of implantation, decreasing at 90, 70, 50 and 0% at respectively 6, 9, 12 and 18 months of implantation [48]. Three-dimensional printing technology could also be used as a solution for cranioplasty procedure [50].

#### 3.5.3. Poly(ε-Caprolactone) (PCL)

Polycaprolactone (PCL) is a semicrystalline polymer of the aliphatic polyester’s family. It is biodegradable and highly compatible with osteoblasts, with a slow degradation rate that preserves its mechanical feature, with good degradation and resorption kinetic that make it suitable as a scaffold in bone tissue engineering application. It is also a polymer less expensive than other biodegradable polymers such as PLA, PGA and their copolymers [51,52,53]. As reported in the literature [53,54], it is a suitable polymer that could be modeled by using computer technology. In particular, as reported by De Santis et al. [54], ink-jet printing (IJP), fused deposition modeling (FDM), laser sintering (LS) and stereolithography (SLA) represent the main additively manufactured (AM) technologies employed for the fabrication of a synthetic structures (biodegradable polymer-based scaffolds) for cranioplasty, with a 3D scan of cranial defects. The technique employed to design the 3D skull and convert it into a 3D virtual model is named reverse engineering (RE) [54]. The combination of RE and AM technologies gives the possibility to fabricate and mold the skull part. PCL and PLA and poly(lactic-*co*-glycolic acid) (PLGA) are the most conventional polyesters manufactured by FDM technology [54]. In particular PCL is easy to mold due to its lower melting temperature (60 °C) compared to the other polyesters. An experimental procedure for design and fabricates 3D porous structures using a commercially available 3D printer (Creality3D Ender-3 PRO) on PCL and PLA was reported by De Santis et al. [54]. PCL in combination with PMMA was further studied [54,55].

Interesting work was reported by Shi et al. [56] on the use of an experimental electrospun PCL–gelatin hybrid membrane planted for preventing adhesion formation and facilitating subsequent cranioplasty. In particular they prepared poly(e-caprolactone)-gelatin (PG) nanofiber membranes with different PCL–gelatin ratios, which were characterized in terms of architectural features, mechanical properties, cell barrier functions, in vivo degradability, biocompatibility and antiadhesion function [56]. The mechanical strength increased by increasing the PCL content while an increase in gelatin content resulted in an enhancement of cell adhesion, proliferation and acceleration of the biodegradation rate [56].

#### 3.5.4. Polyetheretherketone (PEEK)

PEEK, together with PMMA, is one of the most interesting materials used in medicine field such as in spine surgery, orthopedic surgery, prosthodontics surgery, maxillo-facial surgery and cardiac surgery and more recently in cranioplasty [5,10,57,58]. It is a semicrystalline thermoplastic aromatic polymer, chemically inert, which can be easily modelled with a smooth surface and accurately incorporated during cranioplasty, with the aid of 3D printing technology [28,59]. Computer-aided design and manufacture (CAD/CAM) is an emerging technology used actually by surgeons for the head reconstruction with PEEK, reducing surgical time and blood loss [28].

PEEK implants are translucent to X-ray, are nonmagnetic, do not conduct temperature like metallic implants, with strength and elasticity (3–4 GPa) comparable to bones but, at the same time, less dense and with lighter weight [28].

Zhang and collaborators [57] compared PEEK with the most common materials used for cranioplasty such as autologous bone, titanium and PMMA, showing greater performance such as high strength, high toughness and compatibility with skull. Despite these advantages, PEEK implants are very expensive and present scarcity in osteo-integrative properties [57]. To overcome these problems, PEEK can be modified by incorporation of other materials. In particular nanoscale coating of PEEK with bioactive apatite and production of bioactive PEEK nanocomposites was considered, as fully described and reviewed by Zhan et al. [57].

For large format cranial implant another similar polymer was investigated: the polyetherketoneketone (PEKK), approved by the FDA. It is produced by Oxford Performance Materials (South Windsor, Connecticut), with properties similar to the surrounding bone, especially in regard to elastic modulus [18,60]

**Figure 6:**
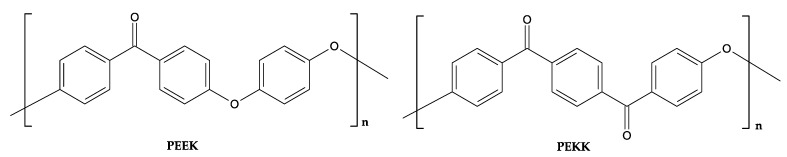
PEEK and PEKK chemical formulas.

The corresponding PEEK and PEKK formulas are reported in Figure 6:

#### 3.5.5. Polyethylene (PE)

PE is an interesting material in the cranioplasty field. It is easy and quick to implant and shows to facilitate an early vascularization [61,62,63]. The infection rate is small [28]. As bioinert material, it does not promote tissue ingrowth and regeneration, but to promote a more consistent bioactive role it could be covered with bioactive materials. The most common form of PE for cranioplasty application is as porous mesh [64,65,66]. As reported by Gosain [37], porous PE, MEDPOR by Stryker (Kalamazoo, Michigan), shows bone and soft-tissue ingrowth, bone and soft-tissue fixation to the surrounding bone, vascular and soft-tissue in- growth into its pores by 1 week, and bone ingrowth by 3 weeks. It can be molded and fabricated using 3D technology.

A similar polymer, i.e., polypropylene (PP) polyester (Knitwear), has been investigated for large format cranial implants [37].

## 4. Future of Cranioplasty

An interesting comparison of cranioplasty/craniofacial implant materials, regarding infection risk, surface form and tissue attachment was presented by Kwarcinski and collaborators [28]. When taking into consideration the infection rate of different biomaterials used in medicine, it is fundamental to compare and understand the material characteristics such as properties, biocompatibility, mechanical performances, bioactivity, chemical resistance, handling and so on. Autologous bone is of course considered the best solution for urgent patient implant but often direct reimplantation of the bone flap is not feasible and the storage requirements (cryogenic or subcutaneous) not always are possible to realize. Several synthetic materials were therefore considered, due to the progress of technology in engineering and medicine fields. PMMA, a medically accepted thermoplastic material, shows high biocompatibility but its curing process followed to prepare the cranial prothesis, is highly exothermic, reaching a temperature of 70–120 °C. This problem is overcome through presurgical plate fabrication or by intraoperative molding external to patient (named templating process). Titanium is considered a good alternative in the field of cranioplasty due to its biological inertness, favorable strength-to-weight ratio and favorable cosmetic and functional outcomes [28]. Nevertheless, despite its Young’s modulus is lower in respect to other metals, the value is higher than that of natural bone, leading to a lessened, but still present, risk of stress shielding. Titanium implants could be too prefabricated, but with increasing operation time and cost. In an alternative to the metal component, PEEK, a linear biocompatible thermoplastic, presents a good balance between strength and rigidity, reducing stress shielding risk [67]. However, PEEK is a hydrophobic material and consequently it does not bind to tissue. Despite a coating that could be used to overcome this problem, uncoated PEEK was used, with the disadvantage in achieving stable fixation in a short time. Polyethylene, a thermoplastic polymer, have recently found application in medicine, due to the possibility of tailoring its physical properties, to be utilized in a broad range of applications [68]. As cranioplasty material it is porous, semi-rigid, strong and flexible. For certain applications, where a higher load is required, the porous structure influences the mechanical properties.

The material characteristics became important for reducing the risk of implantation. Materials that promote vascularization and bone tissue regeneration are preferrable to other materials. Materials porosity play a key role, related of course to the physical structures of the implants and the method of production became an important step to avoid alteration of these physical properties, minimizing implant infection risk. A pore size of about 100 μm is requested to regenerate bone tissue [69]. It was noted that both prefabricated and template methods of implant product could minimize potential structural non-homogeneity and difference in surgical time between surgeries. Additionally, fit, form and function could be now controlled by computers, avoiding further errors coming from hand manipulation. This is the case of materials such as PMMA, PE, titanium mesh and so on [60].

Another important consideration is the relationship between implanting characteristics and patient profiles. Polymers offer the possibility of well modulate shape, size, strength, elasticity and functionality of implant graft materials, and fabrication and implantation cost. Using 3D printer technology is possible to produce a patient-specific cranioplasty implant [53,70], especially when polymers are used because they could be well tailored to the shape of complex craniofacial defects.

**Figure 7:**
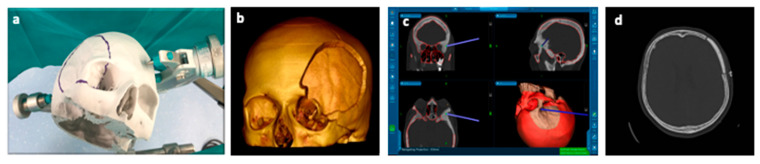
(**a**) Phantom model for virtual craniotomy; (**b**) 3D HR CT-scan of Phantom; (**c**) stealth navigation station; (**d**) post-operative axial CT-scan.

These 3D printing technologies are already used in clinical practice. One example is the one-step procedure that consists of a single surgical operation comprising a resection and reconstruction of cranial defects for lesions involving the skull base [71]. An example is reported in Figure 7:

This procedure takes advantage of virtual 3D skull modelling techniques (Phantom model) (Figure 7a) by acquiring the patient’s high resolution (HR) CT-scan (Figure 7b) and creating a tailored cranioplasty, which reproduces an accurate reconstruction of the bone defect. To achieve better results modern stealth navigation assists the surgeon to perform the craniotomy and match to the custom-made cranioplasty (Figure 7c,d) [20].

## 5. Conclusions

The ideal material for cranioplasty has to be strong, easy to shape, not expensive, with a low rate of infection and radiolucent, biocompatible, porous, firm and stable, in order to provide the greatest advantages to the patients. During the past decades, different materials have served as bone substitutes, alike derived from biological products, others synthetic. All those materials show advantages and disadvantages and consequently should be chosen selectively. Despite metal having been used for cranioplasty since many years ago, the use of autologous bone graft is preferred for reducing the rejection process by the host. It is often preferred when bone defects are not too large otherwise the quantity of available autologous bone might not be enough. The risk of infection, absorption and reduced strength have focused the attention versus synthetic materials, with the desired porosity and the desired wider vascularization. Between the synthetic materials, PMMA, alone or in combination with other materials like titanium, shows excellent tensile strength. Despite its fracture susceptibility and infection rates, it is one of the most extensively used material. PEEK shows the great feature to be perfectly modelled by the use of 3D printing technology, designing specific implants for patients’ craniotomy defects. However, currently, biodegradable natural and synthetic polymers gained even more interest by surgeons, for bone reconstruction. Polymeric implants could be useful because it is possible also to eliminate the additional operation required to remove metal implant and, usually, they do not interfere with therapeutic or diagnostic imaging methods. A third generation of engineered biomaterials, not discussed in this review, is being developed, with the characteristic to encourage cellular responses at a molecular level, in order to promote and accelerate osteogenesis. Such materials will be manufactured with genetically modified cells capable of enhancing bone repair, surpassing the current transplanted autologous standard procedure. This topic could be the object of our future study or by other research groups.

## Data Availability

Not applicable.
